# Osmotic Action

**Published:** 1867-06

**Authors:** H. R. Noel

**Affiliations:** Professor of Physiology.


### ARTICLE IV.

*Osmotic Action.* A Lecture delivered February 18, 1867, at the Baltimore College of Dental Surgery. By H. R. Noel, M.D., Professor of Physiology.

(Concluded.)

*Law III.* Affinity of fluids for each other, we have assumed as being a plain practical fact, as the one is acid and the other alkaline, and therefore the conditions of a current through a membrane are obtained. Each fluid, as it enters the pores, passes to the other side and is dissolved away by the fluid bathing that side, but from previous statements we conclude that the current is stronger from the canal to the vessels, so the circulating fluid takes up and rapidly bears off the contents of the canal. Should the affinity of the two fluids be very strong, but that of one of the fluids for the membrane be very weak, the current is in the direction from the fluid of strong affinity for the membrane to the one of less affinity; or in other words the fluid having an affinity for the membrane, enters the pores at once, and is dissolved away by the other, as fast as it reaches the opposite side; while the current from the fluid of small affinity for the membrane is weak and slow, as in the case of alcohol and water in Lardner's experiment. This may partially explain why alcohol creates such intense thirst—why the victim of a debauch indulges in such copious draughts of water. Theoretically we would say that alcohol has a low endosmotic power, as the affinity between it and the membrane is so small; but its affinity for water causes a flow from the blood to the canal, thus inspissating the blood and causing thirst.

Again after being diluted and slowly absorbed into the vessels, it may still, if not at once burnt off, remain active and cause a flow from the soft structures into the veins and vessels, thus distending them (as was the membrane in Lardner's experiment), and increase the activity of emunctories, as the kidneys and skin, while the soft structures surrounding may be actually inspissated. We could therefore have a perspiring skin—active kidneys—distended veins and yet thirst; for the solid tissues are robbed of their water, or rather of the fluids bathing them by this unnatural perversion of the regular order.

The motion of fluids determines current, other things being equal, in the direction of the most rapid current, the permeating fluid being carried off as fast as it reaches the opposite side, blending with the flowing stream. Here of course the rapidly flowing blood determines the current from canal to vessel, and we could scarcely imagine a sufficiently rapid peristaltic movement of the intestine to cause a change of current to the canal. Other causes may and do produce this reversal of current, but motion is not one of these causes from the very nature of the case.

Writers explain this upon the hydraulic principle of Venturi, viz.: If to the undersurface of a tube containing a fluid flowing through it, another tube be attached, this latter tube terminating in a reservoir, the fluid in the reservoir will rise through the tube to the flowing current, and the reservoir be emptied. Should the fluid be flowing in a conical tube, and in the direction of the larger end, the fluid rises more rapidly and the reservoir is sooner drained, than if the current was flowing in the opposite direction, &c., to the smaller end of the tube.

This principle obtains in full force, in the problem before us, for the digested mass is moving slowly in the canal while the nutritive fluids are moving rapidly in the vessels, and from those of smaller to those of larger calibre; that is from capillaries to veins, and from lacteals to thoracie duct.

In diseases of the liver, lungs, heart, &c., where the circulation is impeded or retarded ; and where the abdominal vessels are distended, this law may cease to act normally, and even be reversed. We may have deficient and tardy absorption; or even serious diarrhœa by congestion of the portal system, reflex congestions of intestinal veins, perverted action and accumulation of fluid in the canal. Dropsies may occur from a similar course, and we are all familiar with swollen feet as a symptom of severe cardiac disease, or pulmonary trouble.

*Law V.* Pressure also determines the current, and as here we have the muscular walls of the digestive tract in continued action, rythmical action almost, of course the current would be from canal to vessels. The strong, firm, regular contraction of the muscular walls upon the mass of digesting material greatly facilitates its absorption. In atony of the muscular coat you would expect deficient absorption, for one important element in the osmotic process is wanting. Abuse of narcotics has the same influence, for the deadened nerves refuse to respond to their normal stimula, and the physicoreflex action of the spinal cord, which regulates the peristaltic movements of the canal fails to be exercised.

Whatever produces muscular atony, whatever deadens the nerves, may cause deficient absorption, indigestion, dyspepsia, &c.

*Law VI.* From an acid to an alkaline fluid, would be from canal to vessels, and therefore the excessive use of alkalies in any form might not only impair digestion proper, but even bar absorption by interfering with this law of osmotic action. Perhaps the use of mineral acids, so popular now in treating typhoid, and typhus fevers, may have some bearing upon this principle, and their antiseptic and digestive powers be still further enhanced by the fact that absorption is also promoted. Certainly the muceous membrane and system generally respond kindly to their exhibition.

But this is not the only instance of motion from an acid to an alkaline fluid, that the body offers us.

Recall for a few moments the discussion of the "Essential Nature of Muscular Action," as given you in a previous lecture, and you will at once perceive how, theoretically at least we can apply this law of current from an acid to an alkaline fluid. The acid muscle juice bathing the fibrillæ, fibres and fasciculi, the alkaline blood of the capillaries taken together, give us the conditions not only for osmotic action, but also the condition of a voltaic circle; conditions of chemico-electrical phenomena.

Assuming that acid muscle juice is an effete product:—result of disintregration, in the descending metamorphosis, and therefore passing rapidly from the dominion of vital to that of purely chemical forces, we can at once appreciate the resort to chemical and physical forces in the osmotic disposition of it. Possibly in the thorough economy of nature, the absorbents may take up, and the lymphatic glands elaborate a few of the elements until sufficiently vitalized to go once more the round of the circulation; and subserve the purpose of nutrition, but the muscle juice as a whole, we none the less consider to be intrinsically effete. Yet in apparent contradiction of this law, the alkaline blood supplies the fibrillæ with plasma and oxygen; the means of destruction and of repair at the same time, so here are two osmotic currents. Perhaps the powerful affinities and forces developed in the chemico-vital processes of nutrition and assimilation, may be sufficiently strong either to equalize the two currents, or to bring only nutritive elements and oxygen through the membranes as neutrals, and destitute of any affiliation as to chemical reactions, with either fluid. The homogeneous, delicate, thin membranes of the capillaries and the sarcolemma, have other and higher functions, therefore, than the mere physical one, of protecting, defining and limiting.

*VII. Law.* Activity of osmosis increased by temperature. This is true of almost every act performed in the animal mechanism, for Heat is one condition and essential of life, and all phenomena of life, even those apparently involving principles purely physical or chemical, are manifested through vitalized structures, and must therefore be dependant more or less upon heat.

The physical and the vital forces are so blended in all the phenomena of life, their laws and conditions so inextricably interwoven that to isolate each—to define the domain of each, is beyond our finite intellects. Man may, in course of developement, be able to examine, measure and classify all of the purely physical; but he has not yet reached that stage, and even should he ever reach it, the "essential nature of vitality" will be still beyond him. In applying the physical principle involved in osmosis, to organized structures, we leave at once the field of rigid mathematical reasoning and enter that of the speculative.

Dr. T. K. Chambers gives another condition as being essential to rapid and complete osmosis; and it is probably the one which should have first claimed our attention. It is "a clean healthy mucous membrane." A membrane covered with tough, tenacious mucus is in the worst possible condition for the exhibition of osmotic phenomena.

Hence cathartics may often be really, good promoters of osmosis, by sweeping clean the digestive tract and thus bringing the fluids in closer approximation; besides exerting a remedial influence upon the vital powers of the tract. They thus enable you to throw more promptly nutritive material and medicinal agents into the vessels.

Chambers gives several cases as examples; cases almost demonstrating as a fact, "that a clean mucous membrane is an essential for normal digestion and normal absorption," Even in anemia if chronic, he will often premise his treatment by a brisk cathartic; sweep the canal free of the tough, tenacious clinging mucus, "clear the decks for action," and then, by generous diet regularly administered at short intervals, and with prompt medication by tonics, iron, &c., throws rapidly into the system the largest possible amount of thoroughly digested material with the best remedial agents, and of course his patients rally as if by magic. As a practical fact, if you wish thorough digestion, thorough absorption, rich blood, red and strong, keep your digestive tract free of this mucus.

Beware of overtaxing your digestive powers, beware of irregular hours and broken meals; beware of habits inducing constipatation and costiveness; beware of severe mental labor soon after meals, or you will violate the laws of normal digestion, violate the laws of normal osmosis also, and the Nemesis of dyspepsia, indigestion, flatulence, and colic will seize upon you and claim a long penalty.

Osmosis, though discussed in relation to digestion and absorption from the canal, has a much wider range both in the animal and vegetable world, than we have been able to give it. All mucous membranes, all synovial and serous membranes, all cells and cell growths, all glandular structures, all muscular action and phenomena, in a word the whole animal economy even in its veriest minutiæ, exhibits more or less of the osmotic action, and depends more or less upon its laws and conditions. Entering into nearly all the phenomena of life, it pervades the system as one of the subtle conditions of existence. Beginning as imbibition and cell action, with the ovum, in the Graafian vessels of the mother, it lasts longer than life, ever active, ever acting ; for when this mechanism, wound up to run its short span of time, ceases from any cause to evolve the conditions necessary for the exhibition of vital phenomena, and vitality is lost; this still retains its power, and as it began when our relations first commenced with our human mother, so is it prolonged until our individuality is lost, and the decomposing wreck, the debris of life crumbles to tbe bosom of our last mother—the earth.

